# Mucocele-like tumor and columnar cell hyperplasia of the breast occurring in a morphologic continuum

**DOI:** 10.1186/1752-1947-2-138

**Published:** 2008-04-30

**Authors:** Oluwole Fadare, M Rajan Mariappan

**Affiliations:** 1 Department of Pathology, Wilford Hall Medical Center, Lackland AFB, TX 78236, USA; 2 Department of Pathology, University of Texas Health Science Center at San Antonio, San Antonio, TX 78229, USA; 3 Department of Pathology, Beth Israel Deaconess Medical Center, Boston, MA, USA; 4 Department of Pathology, Harvard Medical School, Boston, MA, USA

## Abstract

**Introduction:**

Mucocele-like tumor was originally described in 1986 as a benign breast proliferation consisting of multiple dilated cysts lined by cytologically bland, flat to cuboidal cells. Subsequent reports described the coexistence of, including the morphologic inter-transitions between, mucocele-like tumor and a variety of other breast proliferations, including intraductal carcinoma, invasive carcinoma, atypical ductal hyperplasia, and hyperplasia of the usual type. The spectrum of breast alterations characterized by variably enlarged terminal-ductal lobular units lined by variably hyperplastic and variably atypical columnar cells has been the subject of significant discussion in the recent literature. In one scheme, these lesions may be classified into four groups, that is, columnar cell change with and without atypia and columnar cell hyperplasia with and without atypia. Morphologic and molecular observations suggest an association, perhaps in a nonobligate precursor role, between some columnar cell lesions and a variety of other neoplastic lesions.

**Case presentation:**

We describe the case of a 43-year-old woman whose breast tumor contained areas diagnostic of mucocele-like tumor and columnar cell hyperplasia, with morphologic transitions in between.

**Conclusion:**

Our case represents the second broadly similar case that has been reported, and suggests a potential relationship between these two enigmatic lesions.

## Introduction

Mucocele-like tumor (MLT) was originally described in 1986 as a benign breast proliferation consisting of multiple dilated cysts lined by cytologically bland, flat to cuboidal cells [1]. Subsequent reports described the coexistence of, including the morphologic inter-transitions between, mucocele-like tumor and a variety of other breast proliferations, including intraductal carcinoma, invasive carcinoma, atypical ductal hyperplasia, and hyperplasia of the usual type. The spectrum of breast alterations characterized by variably enlarged terminal-ductal lobular units lined by variably hyperplastic and variably atypical columnar cells has been the subject of significant discussion in the recent literature. In one scheme, these lesions may be classified into four groups, that is, columnar cell change with and without atypia and columnar cell hyperplasia with and without atypia. Morphologic and molecular observations suggest an association, perhaps in a nonobligate precursor role, between some columnar cell lesions and a variety of other neoplastic lesions. We describe herein a case that suggests a possible link between MLT and columnar cell lesions.

## Case presentation

During a routine mammogram, a 43-year-old African-American woman with no prior history of breast disease was noted to have several clusters of pleomorphic left breast calcifications. A biopsy of the associated mass lesion was subsequently performed and showed large areas of mucin extravasation and fibrosis but no areas that were unequivocally diagnostic of neoplasia. Mucinous carcinoma could not be excluded based on the pathologic findings, so a decision was made to excise the mass. A needle-localized excision was performed approximately 1 month after the biopsy.

**Figure 1:**
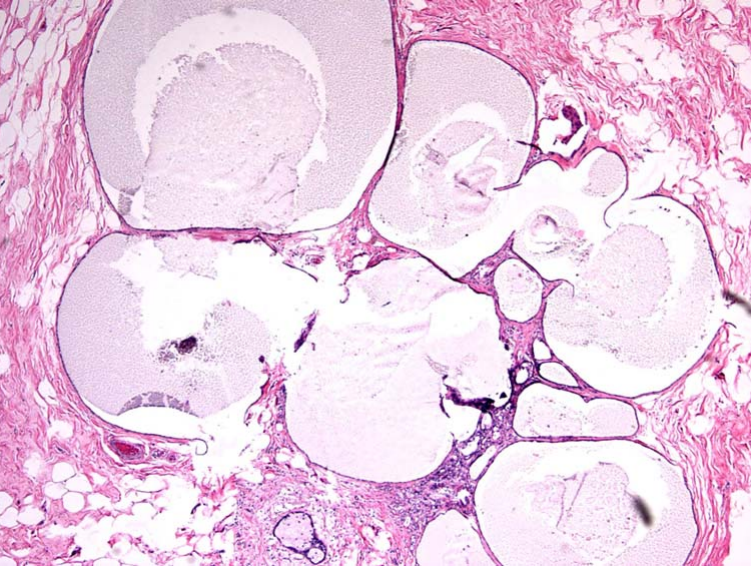
**Mucocele-like tumor**. Hematoxylin and eosin stain, magnification ×80.

**Figure 2:**
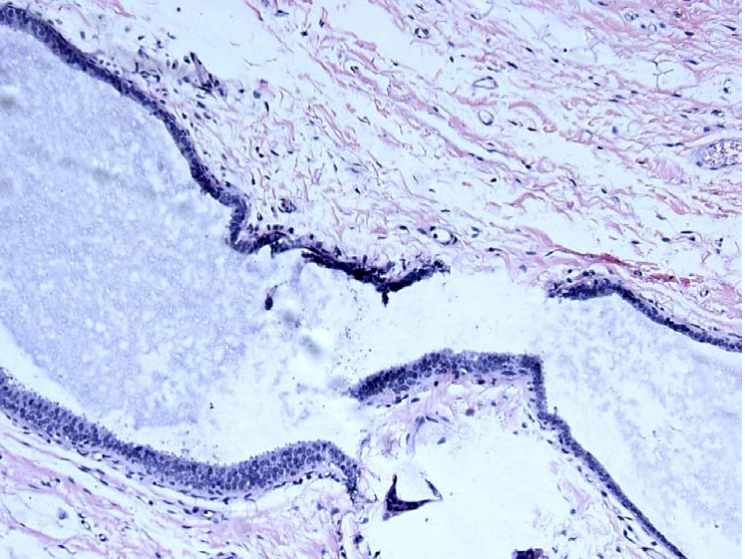
**Within this single dilated duct is a portion lined by hyperplastic columnar cells (single arrow) and flat epithelial cells of the mucocele-like tumor (double arrow)**. Hematoxylin and eosin stain, magnification ×200.

**Figure 3:**
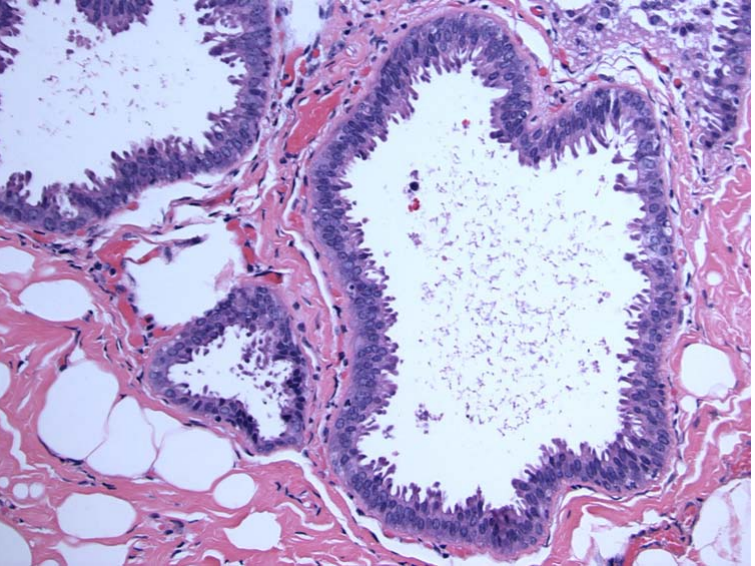
**Columnar cell lesions with atypia (flat epithelial atypia)**. Hematoxylin and eosin stain, magnification ×200.

The excised sample, which measured 10.4 cm, was processed in its entirety for microscopic evaluation. Sections showed diffuse changes diagnostic of MLT, including large cystic spaces lined predominantly by flat attenuated epithelium and variably filled with a lightly amphophilic material (Fig. 1). Occasionally, the lining showed hyperplastic changes. Admixed with the large cystic spaces were tubules with changes diagnostic of nonatypical columnar cell hyperplasia. The latter areas were lined columnar cells, approximately three-cells thick, with variable luminal snouts and no significant cytologic atypia. Notably, some cysts featured an apparent morphologic continuum between the columnar cell areas and the more conventional MLT areas with flat epithelial lining (Figure 2). Transitions were generally 'gradual' within a given duct. In other areas, the lining of the cysts was low cuboidal, that is, within the morphologic spectrum of MLT but suggestive of a transition to columnar cell lesions. A few columnar cell lesions displayed cytologic atypia (Figure 3), but none of the latter showed morphologic transitions with the MLT areas. All areas of the lesion displayed myo-epithelial cells.

Immunohistochemically, the columnar and flat areas had similar Ki-67 proliferative indices, which ranged from 1% to 5% (average 1.7% for MLT, 1.2% for columnar cell lesions without atypia and 3.4% for columnar cell lesions with atypia; mouse monoclonal antibody against human ki-67 antigen, clone mib-1, isotype IgG1-kappa, dilution 1:100, DakoCytomation, Carpinteria, California), and both displayed diffuse immunoreactivity for the estrogen receptor (ER-alpha, mouse monoclonal antibody, clone ID5, dilution 1:50, DakoCytomation). To a large extent, neither the columnar cells nor the attenuated cells of the MLT showed a reduced intensity and/or extent of staining for high molecular weight keratins (HMWK) (monoclonal antibody, clone 34betaE12, dilution 1:50, DakoCytomation), compared with the background ductules. However, in scattered columnar cell-lined ductules, which constituted less than 5% of the columnar cell lesions, which were unassociated with the MLT, and which corresponded to the foci of flat epithelial atypia, the columnar cells showed reduced staining for HMWK. Identical results were found when anti-HMWK was replaced with antibodies to cytokeratin 5/6 (mouse monoclonal antibody, clone D5/16 B4, prediluted, LabVision Corporation/Neomarkers Inc, Fremont, California).

## Discussion

MLT of the breast was originally described by Rosen [1] as a benign proliferation consisting of multiple dilated cysts lined by cytologically bland, flat to cuboidal cells. Subsequent reports described the coexistence of, including the morphologic inter-transitions between, MLT and a variety of breast proliferations, including intraductal carcinoma, invasive carcinoma, atypical ductal hyperplasia and hyperplasia of the usual type [2-4]. Most invasive carcinomas that arise in this setting are of the mucinous type [4], and some authors have postulated a morphologic and biologic continuum between MLT and mucinous carcinomas [3].

The spectrum of breast alterations characterized by variably enlarged terminal-ductal lobular units lined by variably hyperplastic and variably atypical columnar cells has been the subject of significant discussion in the recent literature. In the nosological scheme of Schnitt and Vincent-Salomon [5], these lesions may be classified into four groups, that is, columnar cell change with and without atypia and columnar cell hyperplasia with and without atypia. Lesions with cytological atypia correspond closely with the lesions described as 'flat epithelial atypia', which are recognized in the World Health Organization classification [6,7]. Morphologic and molecular observations suggest an association, perhaps in a nonobligate precursor role, between flat epithelial atypia and lobular neoplasia, tubular carcinoma and low-grade intraductal carcinoma [5-7]. Retrospective analyses have suggested that they may represent a marker of a slightly increased risk for the subsequent development of invasive carcinoma when they are identified in a biopsy [7].

One analysis of columnar cell lesions by comparative genomic hybridization found loss of heterozygosity (LOH) involving 16q, 15q, 16p and 19 in 10 out of 14 cases [8]. This study also showed additional chromosomal abnormalities in columnar cell hyperplasia. However, more recent LOH studies failed to demonstrate any loss of heterozygosity in columnar cell changes without atypia in the three cases analyzed, and LOH in two out of three cases with columnar cell hyperplasia [9]. Hence, the significance, if any, of columnar alterations without significant cytologic atypia remains uncertain.

We have described here a breast lesion which appeared to suggest a potential relationship between MLT and columnar cell hyperplasia given their coexistence and the morphologic transitions between these two lesions. The proliferative indices of the columnar cell lesions and the MLT were heterogeneous but remarkably low, although areas with flat epithelial atypia displayed slightly higher proliferative activity. The low proliferative activity in columnar cell lesions noted in this case is compatible with data reported in a recent study [10]. Both MLT and columnar cell hyperplasia without atypia may simply represent lesions that share derangements in the unfolding of the terminal-ductular lobular units.

This case is somewhat similar to a case recently reported by Coyne [11]. However, in that case, all the cystically dilated spaces were lined by columnar cells, suggesting that the columnar cell lesions were simulating an MLT, as indicated by the author's caption "Columnar cell hyperplasia with intraluminal crystalloids and features of a mucocoele-like lesion" [11]. The case we have described here, in contrast, showed morphologic transitions between areas that are individually diagnostic of MLT and columnar cell hyperplasia without atypia. Nevertheless, these reports suggest that future investigations into a possible link between these two lesions are warranted.

## Conclusion

A single breast tumor that showed a morphologic continuum between mucocele-like tumor and columnar cell hyperplasia is described, which is suggestive of a possible link between these two lesions.

## Abbreviations

HMWK: high molecular weight keratins; LOH: loss of heterozygosity; MLT: mucocele-like tumor.

## Competing interests

The authors declare that they have no competing interests.

## Authors' contributions

OF performed the pathologic evaluation of the case. OF and MRM co-wrote the manuscript. Both authors read and approved the final version of the manuscript.

## Consent

Written informed consent was obtained from the patient for publication of this case report and any accompanying images. A copy of the written consent is available for review by the Editor-in-Chief of this journal.
